# Small bowel obstruction from hollow foreign body ingestion: A case report and brief review of literature

**DOI:** 10.1016/j.radcr.2021.04.025

**Published:** 2021-04-30

**Authors:** Matthew A. Crain, Dhairya A. Lakhani, Ryan Kuhnlein, Aneri B Balar, Susan Neptune, Dan Parrish, Nicholas Shorter, Ayodele Adelanwa, Thuan-Phuong Nguyen, Eyassu Hailemichael

**Affiliations:** a West Virginia University School of Medicine; b Department of Radiology, West Virginia University, Morgantown, West Virginia; c Department of Surgery, West Virginia University, Morgantown, West Virginia; d Department of Pathology, West Virginia University, Morgantown, West Virginia

**Keywords:** Foreign body ingestion, Small bowel obstruction, CT, Computerized tomography, HU, Hounsfield units, US, Ultrasonography

## Abstract

While ingestion of a foreign body by children is common, diagnosis is often challenging, especially when the consumption by a young child is unwitnessed and presenting symptoms mimic other medical conditions. If the foreign body does not pass spontaneously, radiological imaging studies are typically performed, but visualization and identification of the ingested foreign object can be inconclusive, especially when an unidentified mass is radio translucent. Under this circumstance, physicians often have to go on a “fishing expedition”, using exploratory endoscopy and/or surgery to identify and extract the object that became lodged. In this report we discuss a case of a 3 year-old boy who presented with abdominal pain and signs of bowel obstruction. Imaging revealed an ingested “radiolucent” foreign body, masqueraded as soft-tissue mass and enteric duplication cyst, delaying the diagnosis. Systematic shape and density reanalysis of CT and US imaging suggested a hollow object lodged at the terminal ileum. The patient underwent exploratory laparotomy with extraction of a hollow toy “fish”. There is a dearth of literature regarding hollow ingested objects. This case report highlights the importance of systematic density and shape imaging analyses in order to identify and locate hollow ingested objects.

## Introduction

Foreign body ingestion in pediatric patients is common with nearly 70,000 cases reported annually in children under six years of age [1]. While most ingested foreign bodies pass spontaneously, 10% to 20% require endoscopic removal, and 1% need surgical extraction [2]. Diagnosis of foreign body ingestion is often challenging, especially in young children when not witnessed. If the ingested foreign body causes obstruction or injury, presenting symptoms can include abdominal pain, diarrhea, constipation, vomiting, hematemesis, and deceased appetite, which could mimic a wide variety of medical conditions [3].

When symptoms are suggestive of intestinal blockage, differential diagnosis prior to intervention is critical. Radiological imaging is a powerful tool to assess the existence, location, and characteristics of the blockage, including possible foreign body ingestion obstruction [4], [5], [6]. Ultrasonography (US) can be safely used to detect certain ingested foreign bodies, especially if they are superficial, since no radiation is involved [4]. If US does not clarify diagnosis, radiography and computerized tomography (CT) scans are recommended, though visualization often depends on the density of the ingested foreign body in contrast to the densities of the surrounding tissues [4]. Guidelines for imaging ingested foreign bodies indicate radiography prior to CT scans for initial diagnostics since radiographs require less radiation, and a variety of ingested objects are radiopaque, such as stones, metal, and glass, which can often be visualized on radiographs [5]. When radiography is insufficient to determine the nature and cause of the blockage, CT is typically recommended, especially for detection of radiolucent objects, including plastic, water-absorbing objects, and organic foreign bodies, such as wood [4].

Despite the remarkable technological improvements in the diagnosis of foreign body ingestion provided by radiological imaging, many ingested foreign objects remain either not identified or misdiagnosed, and are only determined when extracted by exploratory endoscopy and/or surgery [3], as illustrated by a few recent case reports of small bowel obstruction [8], [9], [10], [11]. We present a pediatric patient with a small bowel obstruction caused by an ingested toy fish that masqueraded as a soft tissue mass on imaging, resulting in delayed diagnosis.

## Case report

A three-year-old, non-verbal male, transferred from a regional hospital to the emergency department of our tertiary hospital, presented with abdominal pain, nausea, vomiting, diarrhea, irritability, and decreased appetite for the past three days. He has a history of Developmental Delay, Atrial Septal Defect, and Autosomal Dominant Familial Focal Epilepsy, controlled on Clobazam and Oxcarbazepine.

**Fig. 1:**
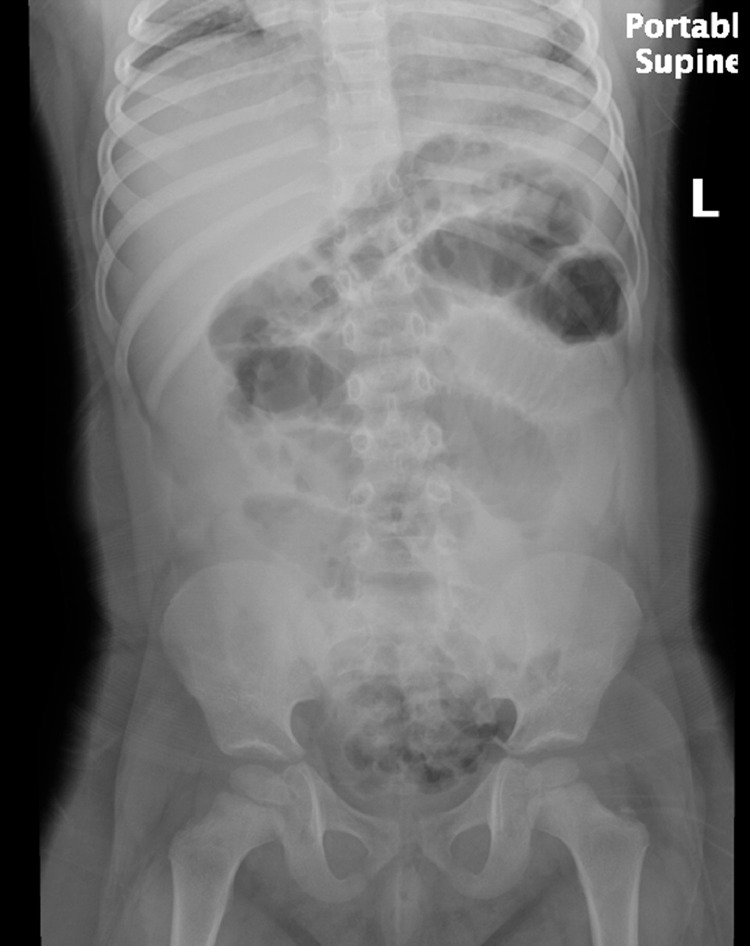
Supine abdominal radiograph demonstrates abnormal gaseous distention of small bowel loops throughout the abdomen, compatible with small bowel obstruction. No pneumoperitoneum or pneumatosis intestinalis.

On arrival to the Emergency Department, he was afebrile and vital signs were stable. Initial laboratory workup including serum lactate was unremarkable. Abdominal radiograph was performed which revealed significant gaseous distention of multiple small bowel loops throughout the abdomen, compatible with small bowel obstruction ((Fig. 1).

**Fig. 2:**
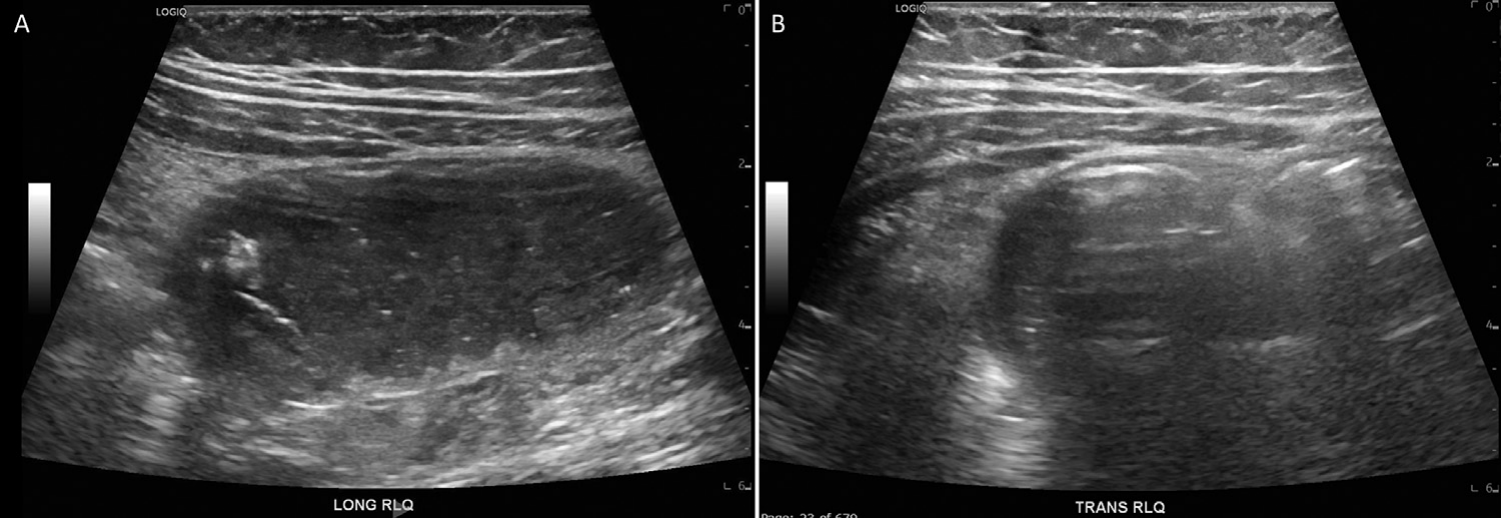
Focused ultrasound examination of the abdomen was performed. A hypoechoic focus was noted in the right lower quadrant, sagittal plane (Fig. 2A) and transverse plane (Fig. 2B). This lesion demonstrated posterior acoustic shadowing. Of note: Appendix was not visualized, perhaps related to projections for surrounding bowel loops.

**Fig. 3:**
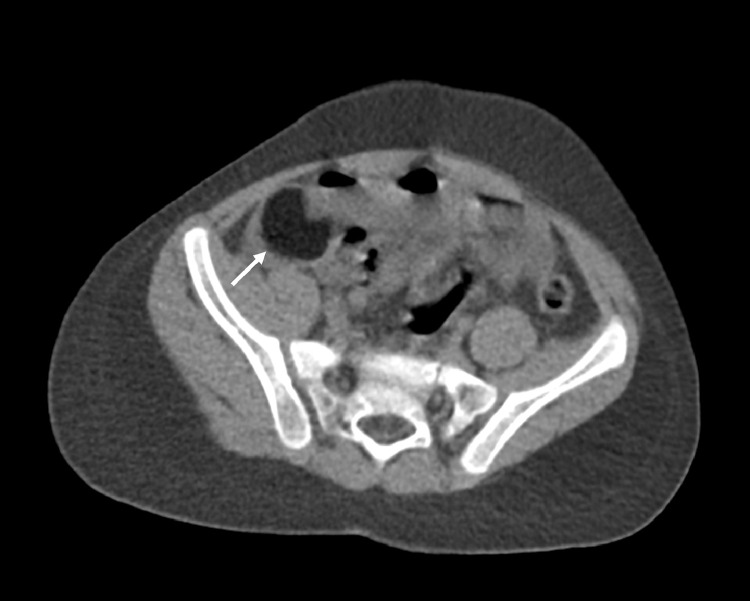
Subsequently, computed tomography (CT) of the abdomen was performed. Axial image demonstrates a 1.8 × 2.9 × 4.3 cm well-circumscribed hypodense mass in the terminal ileum (white arrow), resulting in upstream small bowel obstruction.

**Fig. 4:**
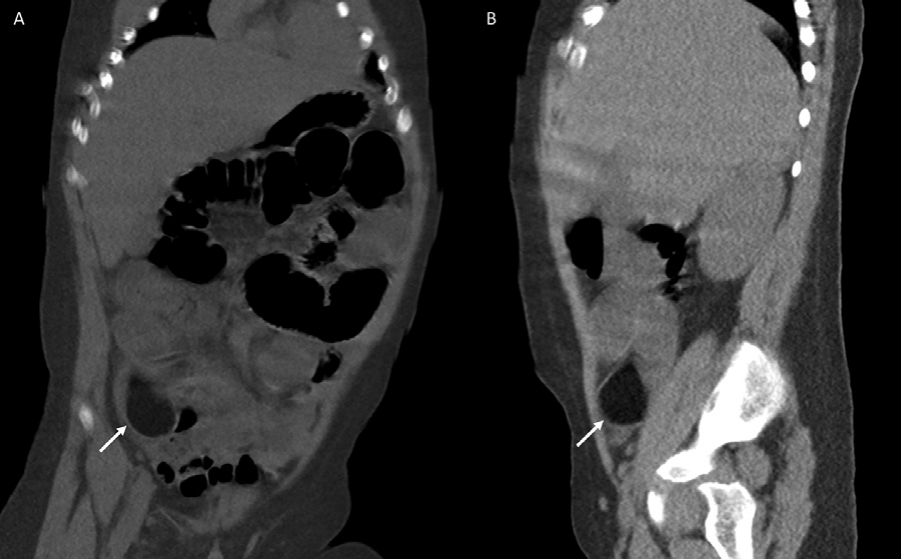
Coronal (Fig. 4A) and sagittal (Fig. 4B) reconstruction images demonstrate a 1.8 × 2.9 × 4.3 cm well-circumscribed hypodense mass in the terminal ileum (White arrow), resulting in small bowel obstruction. No pneumoperitoneum.

Focused ultrasound of the abdomen demonstrated a heterogenous hypoechoic lesion in the right lower quadrant ((Fig. 2). It is to be noted that the appendix was not visualized, perhaps secondary to projections of surrounding dilated small bowel loops. An unenhanced CT of the abdomen and pelvis revealed a 1.8 × 2.9 × 4.3 cm well-circumscribed hypoattenuating lesion in the terminal ileum, resulting in external compression and small bowel obstruction (Figs. 3 and 4A-B). Differentials based on initial imaging included a low-density soft-tissue mass, enteric duplication cyst, and intussusception.

**Fig. 5:**
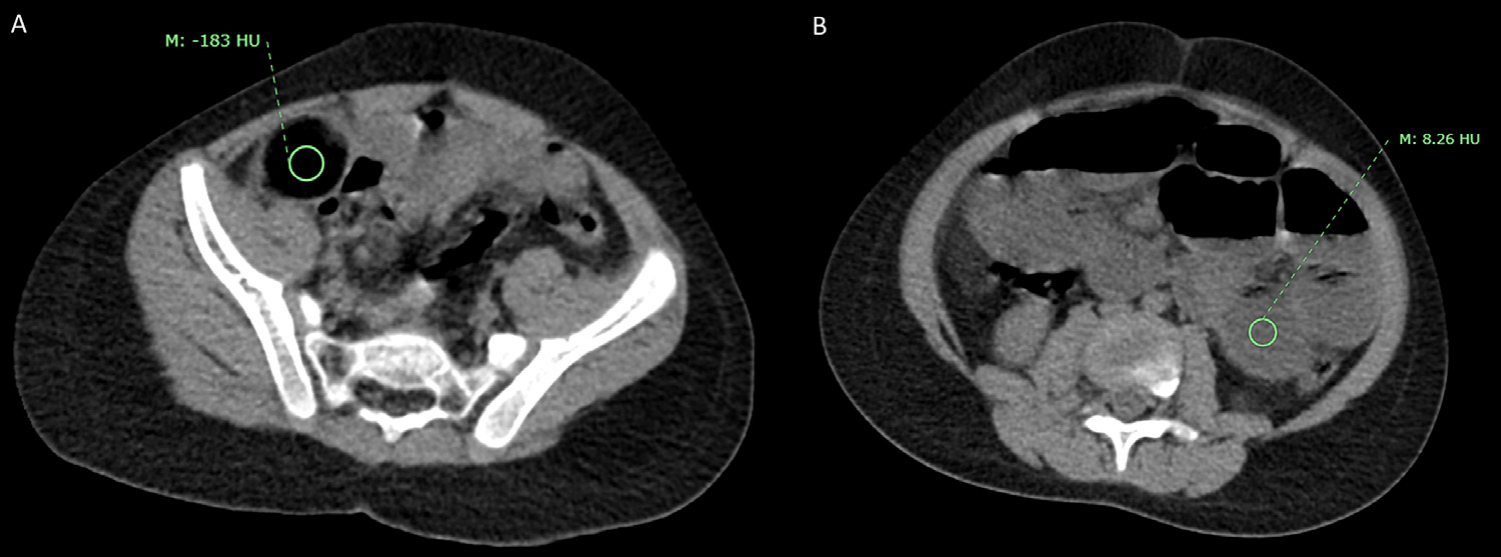
Axial computed tomography (CT) images illustrating Hounsfield Unit of the low-density lesion (Fig. A) as compared to fluid-filled bowel loop (Fig. B).

**Fig. 6:**
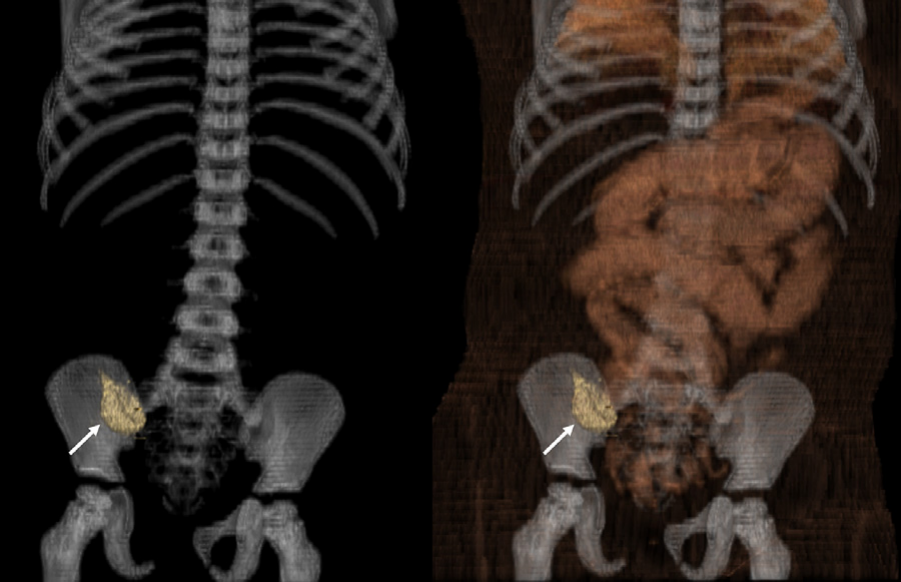
3D reconstructed computed tomography (CT) data, showing low-density lesion (white arrow).

Subsequent reanalysis of the imaging was performed. On further review of previously performed CT, the hypodense lesion had an average density of -183 Hounsfield Unit (HU) (Fig. 5), as compared to the average density of 10 HU of the adjacent fluid-filled bowel loops. Ultrasound examination showed a well-circumscribed focus with almost near-perfect margins; it also demonstrated posterior acoustic shadowing (Fig. 2). The data was favoring ingested radiolucent foreign body as the cause of obstruction in this patient. 3D reconstructions of the CT acquisition are illustrated in (Fig. 6), which further supports the conclusion.

**Fig. 7:**
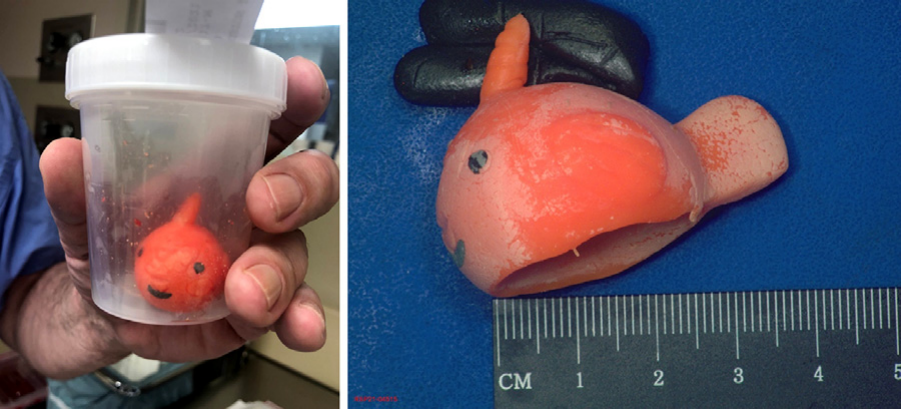
Photograph on the left demonstrates the 3 cm fish-shaped toy removed from the ileum. Following surgery, an incision was made to reveal the hollow nature of the toy, as seen in the photograph on the right.

The patient was subsequently taken to the operating room for removal of the ingested foreign body. Exploratory laparotomy revealed a 3 cm fish-shaped toy from the ileum, which was successfully removed without complications (Fig. 7). Despite the bowel's proximal acute dilation, all functioning appeared normal with minimal blood loss and no expected complications. The patient was monitored in the intensive care unit and had an uneventful recovery. Appropriate parental education was provided prior to discharge.

## Discussion

Foreign body ingestion in pediatric patients is common and has varied presentations [3]. Therefore, state-of-the-art radiological imaging including US, radiographs, and CT scans are often critical to identify the existence, location, and characteristics of ingested foreign bodies. Nevertheless, despite the ability of radiological imaging to visualize certain ingested foreign bodies, accurate diagnosis can be challenging and requires a systematic approach to analyze imaging data. Density variations are a critical component of identifying ingested foreign objects distinct from surrounding tissue. Hounsfield units (HU) are a standardized CT measurement of density, with lower values reflecting less radio-dense materials. The HU scale is centered at 0 (distilled water), with index values of -1000 (air) to +1000 (bones). Common ingested foreign objects include glass (+500 to + 2000 HU), metal (other than aluminum) (>+3000 HU), stone (>+1000 HU), plastic (+100 to +500 HU) [7]. CT analysis includes a density comparison to surrounding tissue, such as fat (-100 HU) and blood (+40 HU) [4]. Therefore, high density ingested foreign objects, such as metal and stone, can often be identified in contrast to surrounding tissue, which is typically lower density. Low density foreign objects, such as rubber balls, are more difficult to identify due to the similarity of density to surrounding tissue. In addition to density analyses, the shape of visualized masses on CT and US imaging, as well as their location, can provide significant clues to the identification of ingested foreign bodies, since man-made and other unusually shaped objects typically have different characteristics than organic tissue formations [6].

This case reflects the critical importance of a thorough and systematic analysis of all imaging data prior to intrusive endoscopy and/or surgery explorations.

There is a paucity of case reports in the clinical literature regarding hollow ingested foreign objects, as reflected in a PubMed search of this condition, which produced only one case [12]. Given the popularity of plastic toys including balls, which are often hollow, their ingestion is likely a common event. Therefore, attention should be given to the potential density and shape of ingested hollow objects, especially for differential diagnostics in the pediatric population. An extremely low-density region in CT imaging, especially when the shape is inconsistent with organic tissue or natural gas pockets, could reflect a hollow air-filled object. A systematic imaging analysis may reduce the need for exploratory "fishing expeditions" and guide endoscopic or surgical extraction.
